# Supplementary material for: Interleukin-22 drives a metabolic adaptive reprogramming to maintain mitochondrial fitness and treat liver injury
Source: Theranostics. 2020 Apr 27;10(13):5879–94. doi: 10.7150/thno.43894 (PMC7254999; doi:10.7150/thno.43894)
Supplement: Supplementary file 1 — Supplementary figures and table. [file thnov10p5879s1.pdf]

Interleukin-22 drives a metabolic adaptive reprogramming to maintain mitochondrial fitness and treat liver injury

Wei Chen[1,2,#], Wenjing Zai[1,3,#], Jiajun Fan[1], Xuyao Zhang[1], Xian Zeng[1], Jingyun Luan[1], Yichen Wang[1], Yilan Shen[4], Ziyu Wang[5], Shixuan Dai[1], Si Fang[1,6], Zhen Zhao[1]*, Dianwen Ju[1]*

[1]Minhang Hospital & Shanghai Engineering Research Center of Immunotherapeutics, School of Pharmacy, Fudan University, Shanghai, China

[2]Department of Ophthalmology, Stanford University School of Medicine, Palo Alto CA 94304, USA

[3]Key Laboratory of Medical Molecular Virology, School of Basic Medical Sciences, Shanghai Medical College of Fudan University

[4]Department of Nephrology, Changhai Hospital, Second Military Medical University, Shanghai, China

[5]Department of Pharmacy, Huadong Hospital, Fudan University, Shanghai, China

[6]Tongcheng Hospital of Traditional Chinese Medicine, Anhui 231400, P. R. China

[#]These authors contributed equally to this work.

*Correspondence to:

Prof. Zhen Zhao, Email: fdmh_zz@fudan.edu.cn

Prof. Dianwen Ju, E-mail: dianwenju@fudan.edu.cn

Minhang Hospital & Shanghai Engineering Research Center of Immunotherapeutics, School of Pharmacy, Fudan University, Shanghai, China

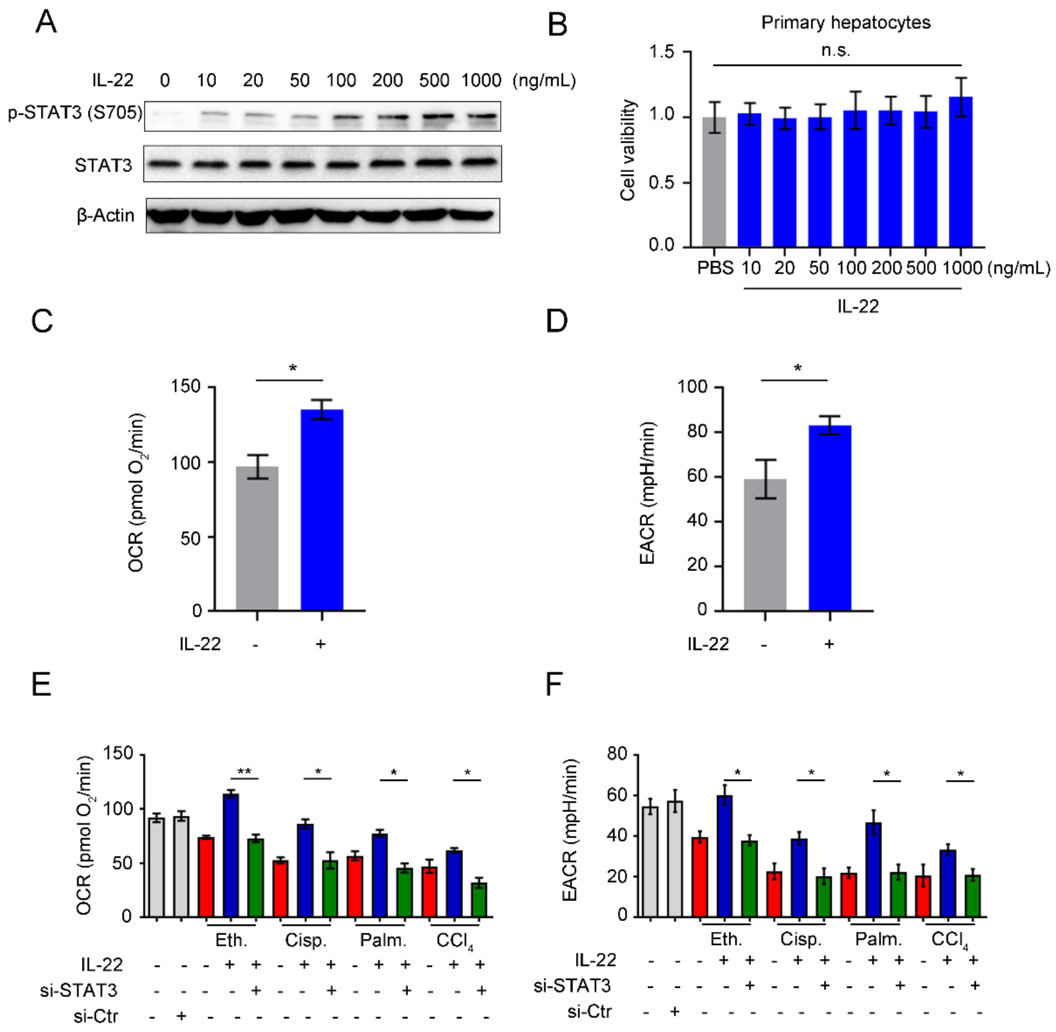

Figure S1. (**A**) Dose-response experiments of IL-22 on hepatocytes. Hepatocytes were stimulated difference doses of IL-22. STAT3 signaling activation in hepatocytes was assessed by western blot analysis. (**B**) *In vitro* cytotoxicity of IL-22 on hepatocytes (n = 4; mean ± SD; n.s., not significant). (**C** and **D**) OCR and ECAR in hepatocytes treated with IL-22 for 24 h (n = 3). (**E** and **F**) Basal OCR and ECAR in hepatocytes at the absence or presence of IL-22, or si-STAT3 for 24 h (n = 3). All data are means ± SD of at least three independent experiments.

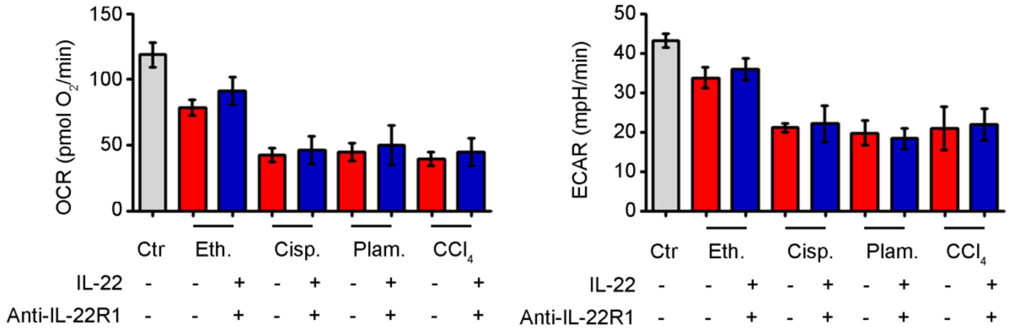

Figure S2. OCR and ECAR in hepatocytes treated with 200 mM ethanol, or 5 μg/mL cisplatin, or 0.25 mM palmitic acid, or 10 mM CCl_4 in the absence or presence of IL-22 and anti-IL-22R1 antibody for 24 h (n = 3; mean ± SD). All data are means ± SD of at least three independent experiments.

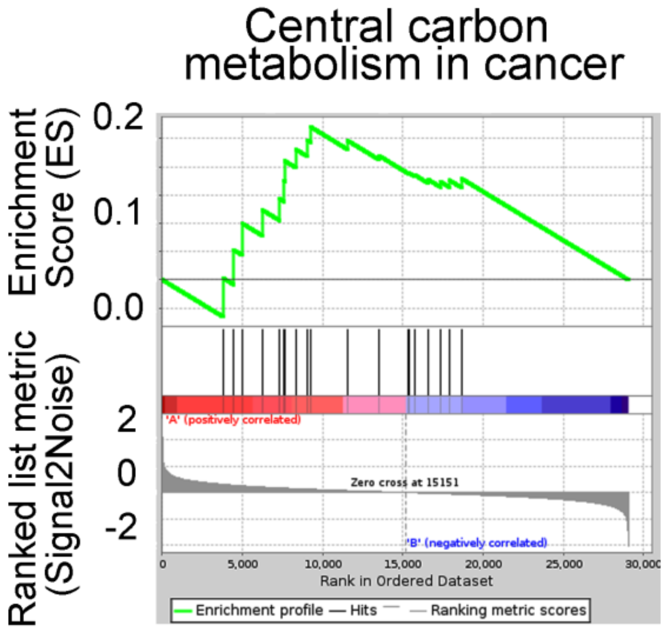

Figure S3. KEGG of central carbon metabolism in cancer in IL-22-protected and -nonprotected hepatocytes.

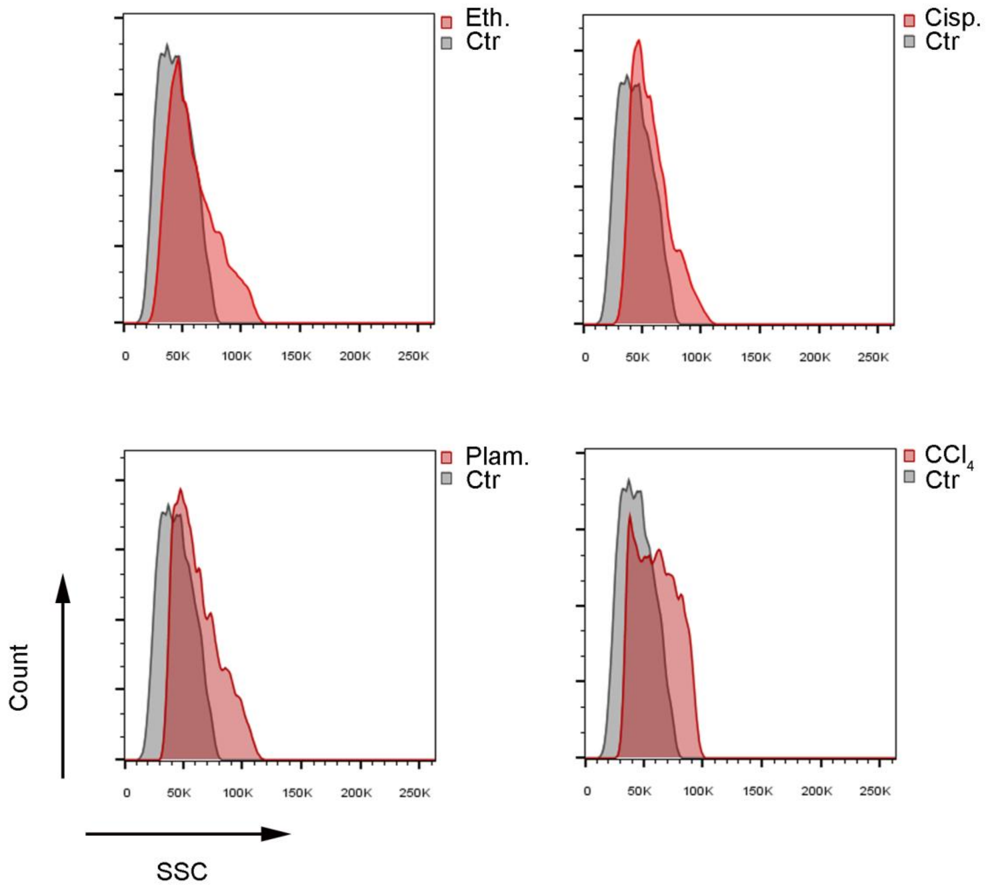

Figure S4. Hepatocytes lead to greater intracellular complexity (reflected by SSC) under injury stress.

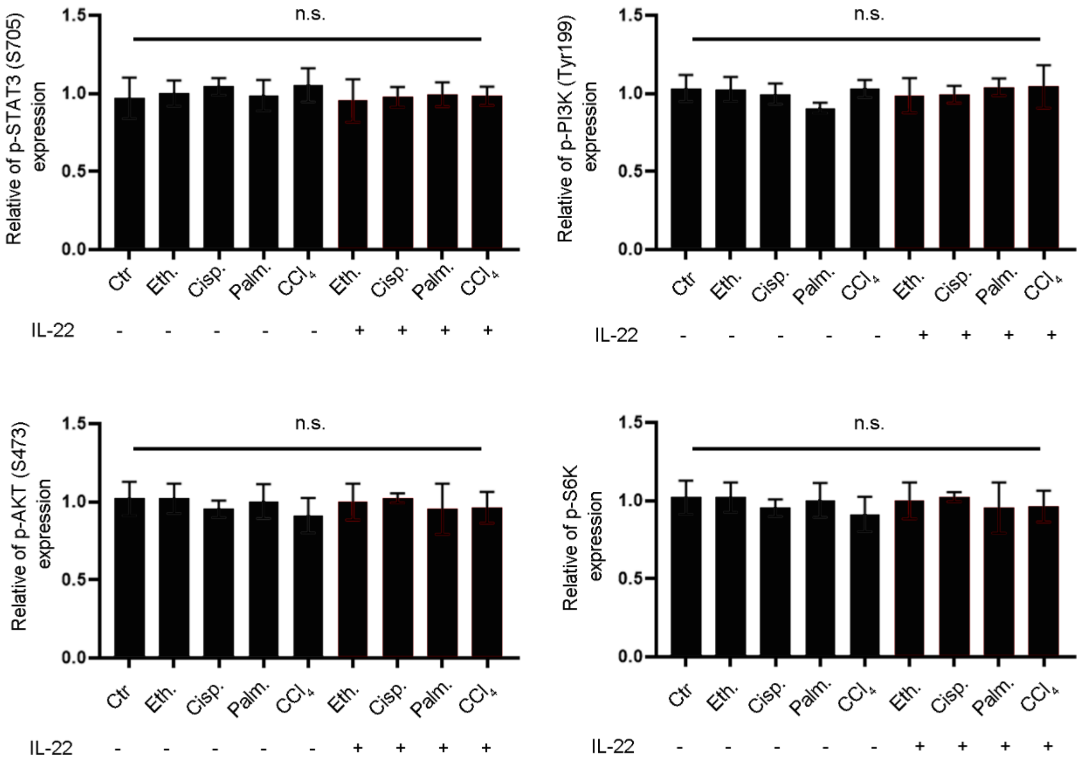

Figure S5. Densitometric values were quantified and normalized to control group (*n* = 3; mean ± SD; ***P* < 0.01, ****P* < 0.001). The values of control group were set to 1.

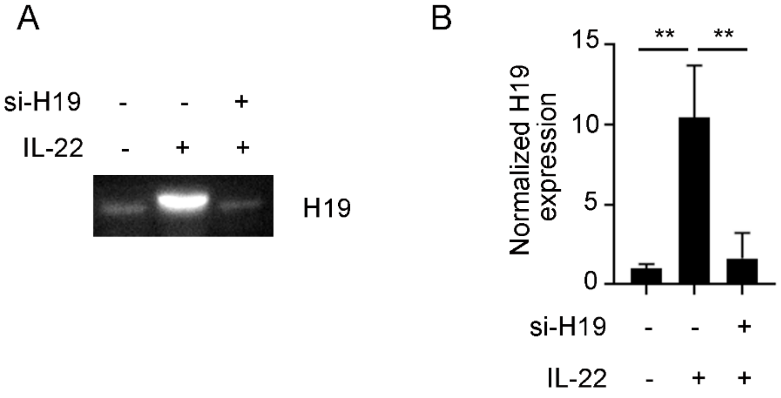

Figure S6. (**A**) Gel electrophoresis analysis of standard RT-PCR of lncRNA H19 and from IL-22-treated hepatocytes. (**B**) Real time PCR analysis suggesting that IL-22 induced lncRNA H19 overexpression in hepatocytes, which could be prevented by

siRNA-lncRNA H19 (si-H19) (n = 3, mean ± SD; **P < 0.01). All data are means ± SD of at least three independent experiments.

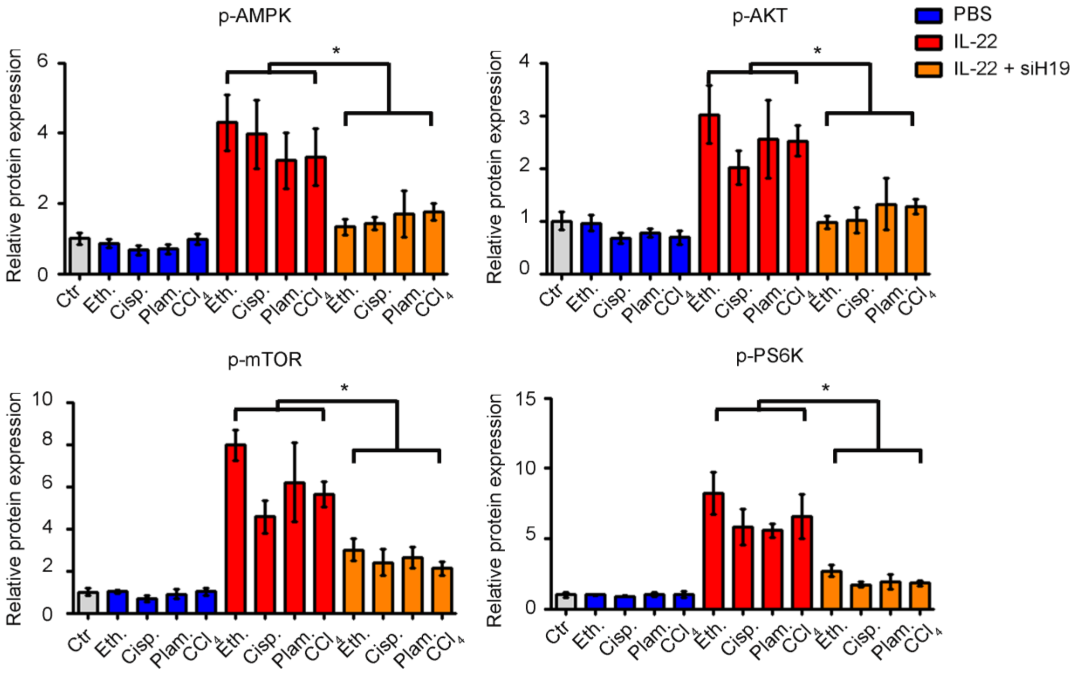

Figure S7. Densitometric values were quantified and normalized to control group (n = 3; mean ± SD; *P < 0.05). The values of control group were set to 1.

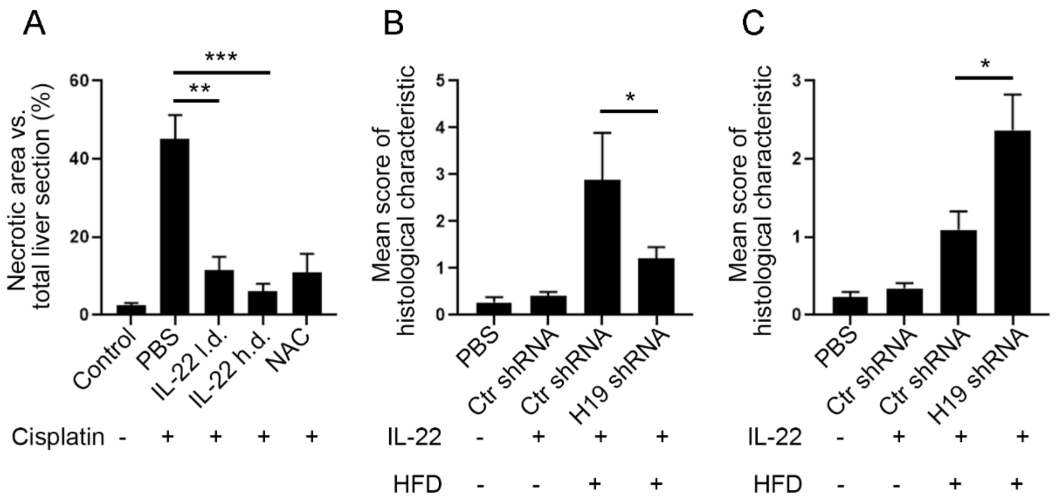

Figure S8. (A) Statistical analysis of necrotic area in H-E stained liver section. (n = 3; mean ± SD; **P < 0.01,). (**B** and **C**) High-fat-diet (HFD)-induced steatohepatitis

activity score.

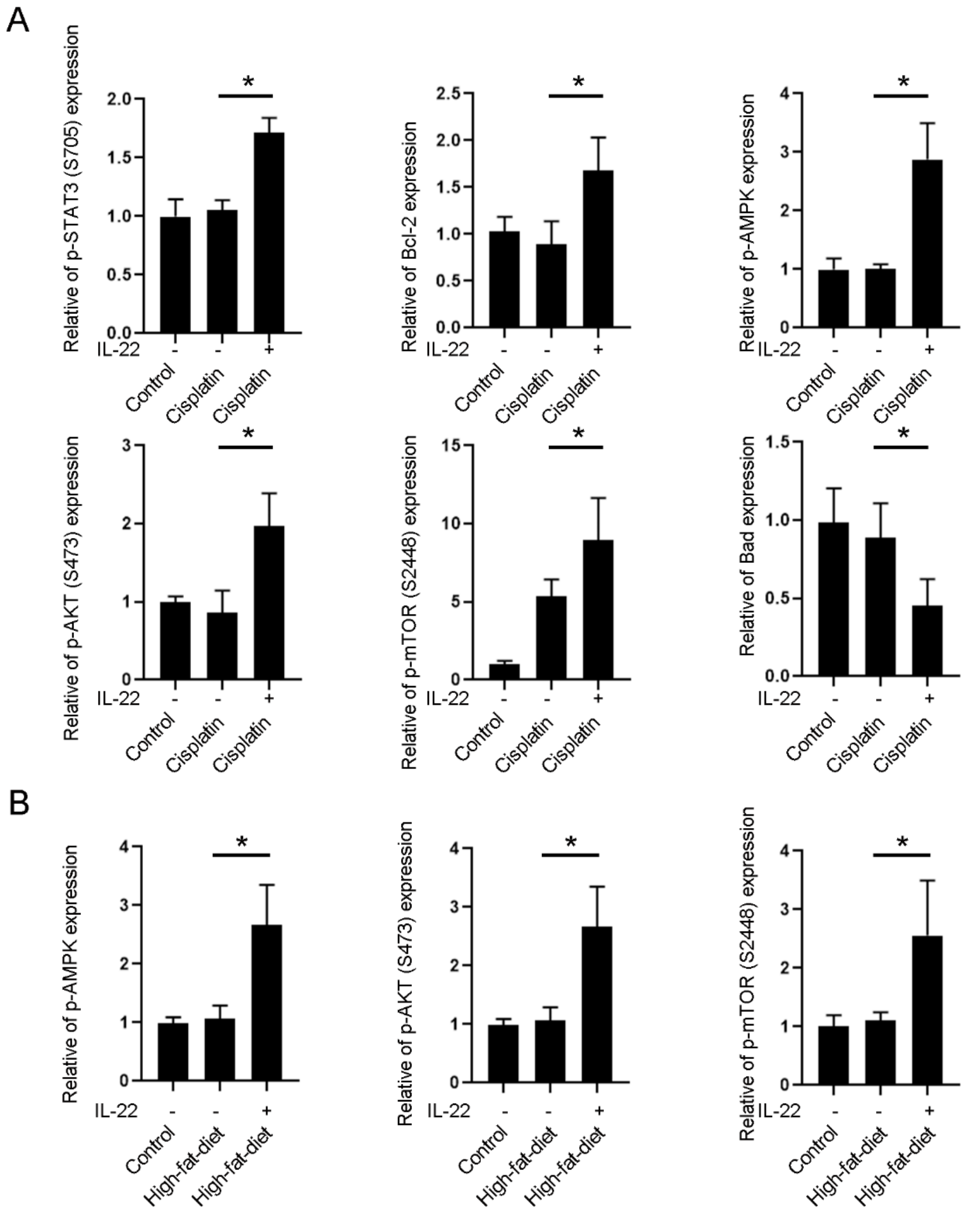

Figure S9. Densitometric values were quantified and normalized to control group (n = 4; mean ± SD; $^{**}P < 0.01$, $^{***}P < 0.001$). The values of control group were set to 1.

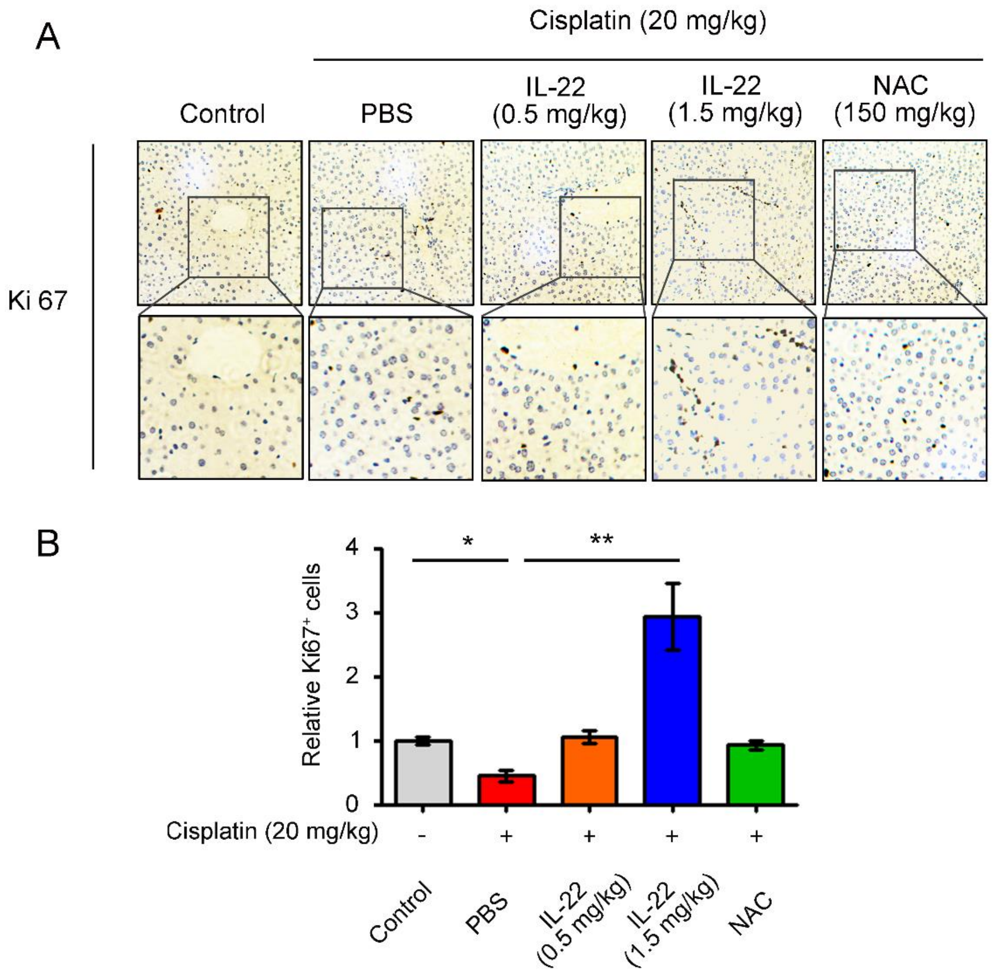

Figure S10. (A) Representative Ki-67 staining images of the liver sections were presented. **(B)** Statistical analysis for the percentage of Ki-67 positive cells (n = 3; mean ± SD; *P < 0.05, **P < 0.01). The values of control group were set to 1.

Supplementary Table 1. List of PCR Primers

**Primer Name**	**Sequence (5'-3')**
GAPDH F1	AACTTTGGCATTGTGGAAGG
GAPDH R1	ACACATTGGGGGTAGGAACA
H19 F1	GACTAGGCCAGGTCTCCAGC

H19 R1	TGACCACACCTGTCATCCTC
IL-6 F1	TTCCATCCAGTTGCCTTCTTGG
IL-6 R1	TTCTCATTTCCACGATTTCCCAG
HK2 F1	GGCGGTTCCGGAAGGAGATG
HK2 R1	GCCAGGCATTCGGCAATGTG
TNF-a F1	AGAACTCCAGGCGGTGCCTA
TNF-a R1	AGTGTGAGGGTCTGGGCCAT
H19 shRNA	GCATGACAGACAGAACATT
